# Comparison of the Stress and Anxiety to Viral Epidemic-9 and SAVE-6 scales among healthcare workers in Peru

**DOI:** 10.3389/fpsyt.2024.1352896

**Published:** 2024-05-01

**Authors:** Bryan Gamonal, Rogelio Quispe-Lizano, Nair Javier-Murillo, André Lapeyre-Rivera, Francisco Perea-Flórez, Víctor Velásquez-Rimachi, Carlos Alva-Diaz, Gilberth Velazco-Gonzales, Oli Ahmed, Seockhoon Chung

**Affiliations:** ^1^ Facultad de Medicina Humana, Universidad de Piura, Lima, Peru; ^2^ Departamento de Investigación, Red de Eficacia Clínica y Sanitaria, (REDECS), Lima, Peru; ^3^ Sociedad Científica de San Fernando, Facultad de Medicina, Universidad Nacional Mayor de San Marcos, Lima, Peru; ^4^ Grupo de Investigación Neurociencia, Efectividad Clínica y Salud Pública (NEURONECS), Universidad Científica del Sur, Lima, Peru; ^5^ Servicio de Neurología, Departamento de Medicina y Oficina de Apoyo a la Docencia e Investigación (OADI), Hospital Daniel Alcides Carrión, Callao, Peru; ^6^ Facultad de Medicina, Universidad Norbert Wiener, Lima, Peru; ^7^ Department of Psychology, University of Chittagong, Chattogram, Bangladesh; ^8^ National Centre for Epidemiology and Population Health, Australian National University, Canberra, ACT, Australia; ^9^ Department of Psychiatry, Asan Medical Center, University of Ulsan College of Medicine, Seoul, Republic of Korea

**Keywords:** test anxiety scale, anxiety, epidemics, health personnel, COVID-19, Peru

## Abstract

**Introduction:**

Peru is a country that has a high incidence of viral outbreaks and epidemics, which is why it is necessary to validate a scale that measures anxiety and stress in professionals who are on the front lines of these events. Therefore, our objective was to validate the Peruvian-Spanish version of the Stress and Anxiety to Viral Epidemics-9 items (SAVE-9) scale and to concurrently compare its validity and internal consistency with the SAVE-6 scale among healthcare workers (HCWs).

**Materials and methods:**

We conducted a cross-sectional study based on data collected from a self-reported survey in paper-and-pencil format between April and July 2023. A total of 203 HCWs participated in the research. We developed a confirmatory factor analysis (CFA) and item response theory (IRT). We calculated Cronbach’s *α* coefficient and McDonald’s *ω* to assess the internal consistency of the scales.

**Results:**

The results show that SAVE-9 (a two-factor model) and SAVE-6 (a one-factor model) provided an excellent fit in the confirmatory factor analysis. Both scales demonstrated strong internal consistency (Cronbach’s *α* 0.85 and 0.86, respectively). Significant correlations were found between the SAVE-9 and SAVE-6 scales and Generalized Anxiety Disorder-7 items scale (*r* = 0.44 and *r* = 0.38, respectively, *p* < 0.001) as well as the Patient Health Questionnaire-9 items (*r* = 0.39 and *r* = 0.35, respectively, *p* < 0.001). The optimal cutoff points for SAVE-9 and SAVE-6 were identified for assessing anxiety, aligned with a GAD-7 score ≥5 points.

**Conclusion:**

The Peruvian-Spanish SAVE-9 and SAVE-6 scales are reliable and valid rating scales to assess the anxiety response of HCWs in response to viral epidemics. Though COVID-19 is diminished, these scales will be useful for other viral epidemics in the future.

## 1 Introduction

The global impact of viral outbreaks is an issue of utmost importance that encompasses areas of public health and scientific research. The sudden spread of these diseases on the planet presents critical challenges in terms of prevention, treatment, and mitigation of their devastating effects (1). Peru is a country that has had countless previous viral epidemics that have marked the country’s health panorama. Among them is the 2009 H1N1 flu pandemic, which claimed many lives, prompted intense national vaccination campaigns, and led to a great economic, social, and health impact (2). In 2016, there was an outbreak of the Zika virus that caused an increase in cases of microcephaly in neonates and cases of Guillain–Barré syndrome in infected people, resulting in a crucial health crisis (3). In 2023, coronavirus disease 2019 (COVID-19) presented an epidemic outbreak between the months of August and September due to the EG.5 strain despite all immunization campaigns (4, 5). Monkeypox has reached nearly 4,000 confirmed cases since its first appearance in June 2023, mainly in Lima (6). Meanwhile, dengue manifested itself in week 20 with more than 400 deaths out of 260,000 cases that occurred, almost 6 times more in 2023 than the previous year (7). Finally, in July 2023, Peru declared a health emergency due to the increase in cases of Guillain–Barré syndrome, which, although its cause is unknown, is associated with bacterial and viral infections (8).

Although viral epidemics affect the entire population, healthcare workers (HCWs) constitute a notably affected group, often experiencing mental health conditions like post-traumatic stress disorder (PTSD), anxiety, and depressive symptoms (9, 10). In the past, SARS and MERS epidemics have been associated with a decline in psychological well-being among HCWs (11, 12). A 2022 report from the Pan American Health Organization (PAHO) highlights the main concerns of healthcare workers which include emotional and financial support, anxiety about infecting family members, problems with relatives of infected people, and changes in usual work functions, factors that could cause alterations in stress and anxiety levels (13).

Therefore, it should be important to specifically evaluate the anxiety responses of HCWs to the viral epidemic to keep an eye on their mental health. In 2021, the scale named Stress and Anxiety to Viral Epidemics-9 items (SAVE-9) was developed by Chung et al. (14). The SAVE-9 scale consists of nine questions that were designed to measure the psychological impact through the evaluation of stress and anxiety during the context of an ongoing viral epidemic aimed toward HCWs. SAVE-9 is a validated tool in many countries such as Russia (15), Japan (16), Italy (17), Korea (14), Germany (18), Turkey (19), and Malaysia (20). The SAVE-6 scale was developed from the SAVE-9 scale and validated in American (21), Lebanese (22), Malaysian (23), and Korean (24) samples to measure the general population’s anxiety response, and it has also been validated in Spain in HCWs (25) and in Peru in medical students (26). The application of SAVE-6 to particular populations was also investigated (27–29), Therefore, it could be suggested that SAVE-6 is a simplified version that does not consider some specific items for HCWs from SAVE-9; thus, it can be used in a broader population. Anxiety and stress in response to viral epidemics represent a distinct dimension of psychological well-being, different from general anxiety or occupational stress. The Stress and Anxiety to Viral Epidemics-9 items (SAVE-9) scale and its abbreviated version, the SAVE-6, were developed to capture this specific response to epidemics. These scales distinguish themselves from other measures by focusing on the unique experiences of HCWs in these crisis contexts. The SAVE-9 or SAVE-6 scales were designed to assess one’s viral anxiety or stress, not just associated with COVID-19 but also with any viral epidemic in the future. Given the situation in Peru regarding viral outbreaks, an important need has arisen and that is to ensure the mental health of health professionals. To achieve this, it is essential to validate a scale that measures psychological disorders arising from epidemic outbreaks in this vulnerable group.

Although the SAVE-6 scale has previously been validated among healthcare workers in Spain and medical students in Peru, its direct assessment in the specific context of Peruvian HCWs facing viral outbreaks has not yet been conducted. In this study, we aimed to validate the Peruvian-Spanish version of the SAVE-9 scale specifically among HCWs in Peru and to concurrently compare its validity and internal consistency with the SAVE-6 scale. This comparison is crucial to ascertain whether the abbreviated version (SAVE-6), which potentially offers greater convenience for rapid practical applications, retains its reliability and validity in the challenging context of viral outbreaks encountered by HCWs in Peru. Moreover, by evaluating both scales, this study seeks to identify which one provides a more accurate measure of anxiety and stress related to viral epidemics in this specific group, addressing both the need for effective assessment tools and the optimization of resources in overstretched healthcare settings.

## 2 Methods

### 2.1 Study design

This study was conducted with a sample of 203 healthcare workers from the Luis Negreiros Vega Hospital, selected through convenience sampling between April and July 2023. This provides a broad basis for analyzing the validity and internal consistency of the SAVE-9 and SAVE-6 scales in an epidemic context. The hospital is a category 2 (II-2) second-level care health facility with the capacity to provide comprehensive outpatient, emergency, and specialized hospital care services for injuries of intermediate complexity (30). Additionally, we reported this study according to the Strengthening Reporting of Observational Studies in Epidemiology guidelines for cross-sectional studies (STROBE) (31).

### 2.2 Participants and procedure

Healthcare workers in Luis Negreiros Vega Hospital who answered “Yes” to the question about participation against viral epidemics, which means that they have ever worked in healthcare during a pandemic at some point in their lives and completed the Peruvian-Spanish SAVE-9 and SAVE-6, PHQ-9, and GAD-7 self-administered scales, were enrolled as participants. Participants were asked to provide information on their demographic variables, including gender, age, department/service, and career. The survey took place in a paper-and-pencil format at their respective workplaces, after agreement with the hospital authorities. The authors administered the survey in small groups (approximately five people) and provided the instructions and all the necessary information related to the research. In all cases, written consent was obtained by means of a note clarifying the purpose of the study and guaranteeing the voluntary and anonymous nature of participation. The sample size was determined based on the recommendation that a range of 200–300 is appropriate for factor analysis (32).

### 2.3 Rating scales

#### 2.3.1 Stress and Anxiety to Viral Epidemic-9 items and SAVE-6 scales

The SAVE-9 scale is a self-report measurement tool designed to evaluate HCWs’ work-related stress and anxiety due to the viral pandemic (14). This scale also includes nine items which can be rated on a five-point Likert scale (0: never–4: always), resulting in a total score ranging from 0 to 36 points (14). The SAVE-9 scale encompassed two main aspects: factor I, centered on “Anxiety about the epidemic” (items 1, 2, 3, 4, 5, and 8, forming SAVE-6) (24) and factor II, addressing “Work-related stress associated with the epidemic” (items 6, 7, and 9, forming SAVE-3) (33). In this study, we adapted and applied the Spanish translation of the SAVE-9 scale developed by Moraleda-Cibrián et al. (25). Additionally, we used the SAVE-6 scale to assess their respective applicability to HCWs. The scale was previously validated among medical students in Peru (26) and HCWs in Spain (25).

#### 2.3.2 Generalized anxiety disorder-7

The GAD-7 is a self-administered questionnaire consisting of seven questions that assess general anxiety, unlike the SAVE-6 and SAVE-9, which are aimed at measuring anxiety in the specific context of an epidemic/pandemic due to a virus, not in any everyday situation. The Likert scale is used to assess every item with four points (0 = never and 3 = almost every day). The total score for the whole scale ranges from 0 to 21. The higher the GAD-7 score, the higher the degree of anxiety indicated. Anxiety was classified based on scores into four categories: minimal (0–4 points), mild (5–9 points), moderate (10–14 points), and severe (15–21 points); experts also recommend using a score of 10 as a cutoff point to identify potential cases of generalized anxiety disorder (34). In this study, the GAD-7 questionnaire with Spanish validation in Colombian HCWs was used (35). Cronbach’s *α* among this sample was 0.86.

#### 2.3.3 Patient Health Questionnaire-9

The Patient Health Questionnaire-9 (PHQ-9) is a nine-item instrument designed to assess symptoms of depression. All items are scored on a Likert scale ranging from 0 (never) to 3 (almost every day), with a total instrument score ranging from 0 to 27. A higher PHQ-9 score indicates greater severity of depressive symptoms (0 to 4 = minimal depression, 5 to 9 = mild depression, 10 to 14 = moderate depression, 15 to 19 = moderately severe depression, and ≥20 = severe depression) (36). The PHQ-9 suggests major depression when at least five depressive symptom criteria are present “more than half the days” over a period of at least 2 weeks, with depressed mood or anhedonia always present (36). In this study, we used the Spanish version of the PHQ-9, which has been validated in the general population in Peru (37). Cronbach’s *α* among this sample was 0.88.

### 2.4 Statistical analysis

Data management and analyses were performed using SPSS version 21.0 (SPSS, Inc, Chicago, IL, USA) and JASP version 0.14.1.0 (JASP Team, Amsterdam, The Netherlands). See **Appendix A** for the complete item content of the SAVE-9 scale and **Appendix B** for the SAVE-6 items, both presented in Spanish. These items reflect the specific concerns and work-related stress experienced by healthcare workers during viral epidemics.

#### 2.4.1 Descriptive statistics

Descriptive analysis was performed to describe the sociodemographic characteristics of the sample (frequency, mean, and standard deviations). Skewness and kurtosis were used to test the normality assumption for each item. Acceptable values range from −1.5 to +1.5, with values between −1 and +1 being excellent (38).

#### 2.4.2 Item analysis

A correlation analysis was performed between the scores of each item and the total score minus the item score, with an acceptable corrected item–total correlation (CITC) greater than 0.30, demonstrating that the item has an appropriate design (39).

#### 2.4.3 Structural validity

Various methods, such as content validity, construct validity, and criterion‐related validity, are used to test the validity of a scale (40). While developing the original form of the scale, Chung et al. investigated the relationship between SAVE‐9, PHQ‐9, and GAD‐7 and found a significant relationship between SAVE‐9 and other scales (14).

To evaluate the structural validity of the Peruvian-Spanish SAVE-9 and SAVE-6, we conducted an assessment of their psychometric properties using both classical and modern test theory approaches. Under the classical test theory approach, Diagonal-Weighted-Least-Squares (DWLS) was used to perform confirmatory factor analysis (CFA) on the SAVE-9 and SAVE-6 scales to verify their factor structure. This method was chosen due to its better fit for ordinal item constructed scales (41–43). For computing CFA, we used the lavaan library in the RStudio interface (44). First, the Kaiser–Meyer–Olkin (KMO) value and Bartlett’s sphericity test were examined to verify sampling adequacy and data suitability for factor analysis. A KMO value above 0.7 and a *p*-value less than 0.05 in the Bartlett’s test are the minimum standards to meet (45). Items with factor loadings of <0.4 are weak, and factor loadings >0.6 are very strong (46). Satisfactory model fit for the factor structure was defined as a −*χ* ^2^/*df* ratio ≤5 (47), standardized root mean square residual (SRMR) value ≤0.06 (48), a root mean square error of approximation (RMSEA) value ≤0.08 (48), and a comparative fit index (CFI) and Tucker–Lewis index (TLI) values ≥0.95 (47). A multigroup CFA analysis (estimation = DWLS) with configural invariance testing (49) was performed to determine whether the Peruvian-Spanish SAVE-9 and SAVE-6 can equally and accurately measure viral stress and anxiety in HCWs across genders (male vs. female) and individuals with depression (PHQ-9 ≥10 vs. PHQ-9 <10). Invariance was assessed utilizing Δ*χ* ^2^ (*p* < 0.05) (50) and ΔCFI (≤0.10) (51). The inclusion of gender in our analyses is grounded in existing literature, suggesting that there may be significant differences in how men and women experience and cope with stress and anxiety (52).

Under the modern test theory approach, the Peruvian-Spanish SAVE-9 and SAVE-6 scales were also evaluated using the graded response model (GRM), a contemporary model for testing polytomous items, of the item response theory (IRT) (53). Item fits were evaluated using S-*χ* ^2^ (FDR-adjusted *p*-values) and RMSEA values. An item is considered a misfit when the *p*-value of the S-*χ* ^2^ is <0.001 (54) and the RMSEA value ≤0.08 (55). The GRM provides parameters for discrimination/slope (*α*) and threshold/difficulty (*β*). The discrimination parameter (*α*) represents an item’s ability to differentiate among individuals with varying degrees of latent trait (*θ*). An item with a higher discrimination parameter provides more information than an item with a lower discrimination parameter (0 = none, 0.01 to 0.34 = very low, 0.35 to 0.64 = low, 0.65 to 1.34 = moderate, 1.35 to 1.69 = high, >1.70 = very high, and + infinity = perfect) (56). The threshold parameters (*β*) indicate a latent trait (*θ*) required to choose a specific response category over another with a 50% probability of selection. Furthermore, scale information curves and item characteristic curves for each scale were computed.

#### 2.4.4 Internal consistency analysis

To test the internal consistency of the Peruvian-Spanish SAVE-9 and SAVE-6 scales, Cronbach’s *α* coefficient and McDonald’s *ω* coefficient were computed for the entire scales, and 95% confidence intervals (95% CI) were provided. A Cronbach’s *α* coefficient/McDonald’s *ω* coefficient above 0.7 indicates high internal consistency, while a value above 0.9 implies redundancy (57). McDonald’s *ω* coefficient takes into account the ordinal nature of the data, and as such, it is recommended for Likert-type item scores (58). Additionally, the internal consistency of the IRT was calculated (59).

Criterion‐related validity was assessed by correlating the Peruvian-Spanish SAVE-9 and SAVE-6 with the PHQ-9 and GAD-7 scales using Pearson’s correlation coefficients. Finally, the receiver operating characteristic (ROC) analysis was performed to explore the appropriate cutoff score of the Peruvian-Spanish SAVE-9 and SAVE-6 scales in accordance with “at least a mild degree of anxiety” by a GAD-7 score of 5 (GAD-7; ≥5) (14).

### 2.5 Ethical considerations

The study protocol was approved by the Institutional Research Ethics Committee of the *Universidad de Piura* (No. PREMED07202213) and the Training Unit of the Luis Negreiros Vega Hospital (No. 718720225024).

## 3 Results

### 3.1 Sociodemographic characteristics

In this study, 203 healthcare workers participated, with 64% (130/203) women, resulting in a male/female ratio of 0.56. The median age of the participants (*n* = 159) was 40 years (Q1: 35; Q3: 46). HCWs were nurses (73/203) and worked in emergency services (51/203) (**Table 1**). The participants’ mean rating scale results are described in **Table 1**.

**Table 1:** Demographic characteristics of the participants (*N* = 203).

Variables	Mean ± SD, *N* (responses %)
Sex (female)	
Male	69 (33.99)
Female	130 (64.04)
Do not want to answer	4 (1.97)
Age (years, *N* = 159)^a^	
20–29	20 (12.58)
30–39	50 (31.45)
40–49	58 (36.48)
50–67	31 (19.50)
Career (*N* = 202)^b^	
Physician	47 (23.27)
Nurses	73 (36.14)
Medical technologist	16 (7.92)
Technical and auxiliary assistant	47 (23.27)
Allied healthcare workers (nutritionist-dietitian, obstetrician, dentist, psychologist, pharmacist, social worker)	19 (9.41)
Main department/service (*N* = 203)	
Department of Medicine	47 (23.15)
Emergency Service	51 (25.12)
Pediatric Service	14 (6.90)
Physical Medicine and Rehabilitation service	4 (1.97)
Surgery Department	16 (7.88)
Obstetrics and Gynecology Service	5 (2.46)
Department of Diagnosis and Treatment Assistance	39 (19.21)
Nursing Service	27 (13.30)
Rating scale scores	
Stress and Anxiety to Viral Epidemic-9 items (SAVE-9)	14.29 ± 6.96
Stress and Anxiety to Viral Epidemic-6 items (SAVE-6)	10.98 ± 5.39
Generalized Anxiety Disorder-7 (GAD-7)	2.29 ± 2.99
Patient Health Questionnaire-9 (PHQ-9)	2.43 ± 3.52

a Forty-four participants did not answer this question.

b One participant did not answer this question.

### 3.2 Item analysis

Item-level descriptive statistics are demonstrated in **Table 2**. Items’ mean scores ranged between 0.87 (SD = 1.02) (item 7) and 2.34 (SD = 1.24) (item 8). Skewness (ranging between −0.25 and 1.22) and kurtosis (ranging between −0.97 and 1.20) are within acceptable values. The CITC ranged from 0.37 to 0.81 which is acceptable, except for item 9 (CITC = 0.24) (**Table 2**).

**Table 2:** Factor structure of the Peruvian-Spanish SAVE-6 and SAVE-9 among healthcare workers (*N* = 203).

Items	Response scale					Descriptive				CITC		Factor loading		
	0	1	2	3	4	M	SD	Skewness	Kurtosis	SAVE-9	SAVE-6	SAVE-9 factor I	SAVE-9 factor II	SAVE-6
**Item 1**	16.7	15.8	36.0	16.7	14.8	1.98	1.26	−0.01	−0.86	0.79	0.79	0.84		0.87
**Item 2**	14.8	21.7	31.5	14.8	17.2	1.99	1.29	0.10	−0.97	0.77	0.77	0.83		0.85
**Item 3**	14.8	17.7	38.4	9.9	19.2	2.02	1.28	0.10	−0.87	0.81	0.81	0.86		0.89
**Item 4**	16.7	41.9	32.5	7.4	1.5	1.34	0.90	0.38	−0.01	0.37	0.37	0.41		0.37
**Item 5**	18.7	44.8	26.6	6.9	3.0	1.32	0.97	0.70	0.43	0.48	0.48	0.54		0.49
**Item 6**	35.5	33.5	24.1	3.9	3.0	1.05	1.01	0.83	0.35	0.38			0.47	
**Item 7**	45.8	31.0	16.7	3.0	3.4	0.87	1.02	1.22	1.20	0.38			0.42	
**Item 8**	9.4	14.3	32.0	22.2	22.2	2.34	1.24	−0.25	−0.82	0.68	0.68	0.75		0.74
**Item 9**	26.6	30.0	28.6	10.3	4.4	1.37	1.13	0.50	−0.45	0.24			0.58	

0 = never, 1 = rarely, 2 = sometimes, 3 = often, 4 = always.

M, mean; SD, standard deviation; CITC, corrected item–total correlation; CID, Cronbach’s α if item deleted; CI, confidence interval.

### 3.3 Structural validity

#### 3.3.1 Peruvian-Spanish version of SAVE-9

**Figure 1:**
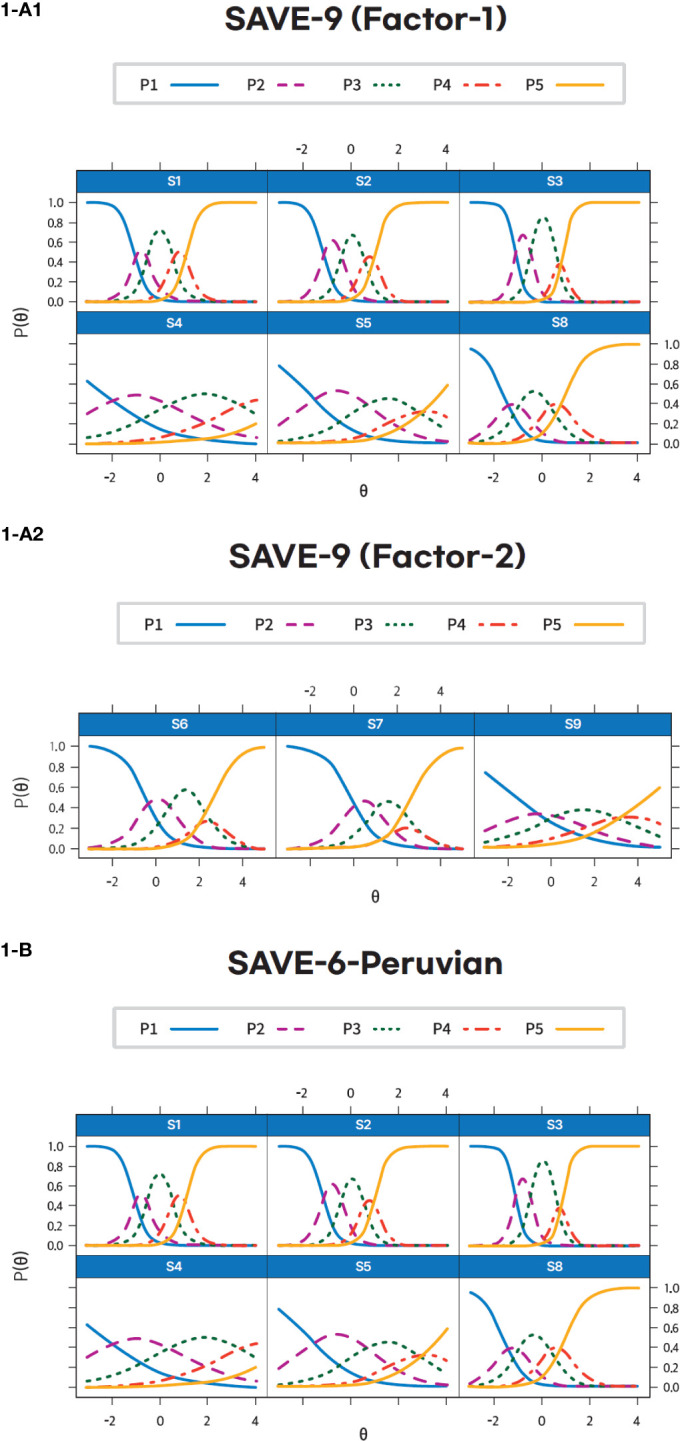
Item’s threshold curves of the Peruvian-Spanish version of the SAVE-9 (Factor 1-**(A1)**, Factor 2 – I**(A2)**) and the SAVE-6 among healthcare workers (1-**(B)**).

Sample adequacy and data suitability were thoroughly assessed prior to factor analysis. The KMO value was 0.86, while Bartlett’s test of sphericity demonstrated statistical significance [*χ* ^2^ (36) = 775.22, *p* < 0.001]. Factor loadings were acceptable to strong and ranged between 0.41 (item 4) and 0.84 for factor I (item 1) and between 0.42 (item 7) and 0.58 (item 9) for factor II (**Table 2**). The CFA suggested that the two-factor correlated structure of the Peruvian-Spanish SAVE-9 had a good fit [*χ* ^2^/*df* = 1.30, CFI = 0.99, TLI = 0.99, RMSEA = <0.001, SRMR = 0.07) (**Table 3**). The multigroup confirmatory factor analysis results demonstrated that the configural model had a good fit (*χ* ^2^/*df* = 0.89, CFI = 1.00) with respect to the general model. The Δ*χ* ^2^ (*p* = 0.294 and *p* = 0.216) and ΔCFI (<0.001 and <0.001) values in the metric and scalar models, respectively, suggested invariance between men and women (**Supplementary Table 1**). In the GRM analysis, both factor 1 and factor 2 items demonstrated a good fit, as evidenced by the S-*χ* ^2^ *p*-values and RMSEA values listed in **Supplementary Table 2**. The slope parameters (*a*) ranged between 0.78 (item 4) and 4.91 (item 3) for factor I and between 0.72 (item 9) and 1.86 (item 6) for factor II. The slope parameters of items 4, 5, and 9 are moderate, items 6 and 7 are high, and the rest are very high. The results for threshold parameters (*b*) indicate that items 4 and 5 require a higher latent trait (*θ*) to endorse response options “sometimes” to “always.” On the other hand, a higher latent trait (*θ*) is required to endorse the response options “often” and “always” for the rest of the items in factor I. Similarly, in factor II, threshold parameters (*b*) indicate that a higher latent trait (*θ*) is required to endorse response options “sometimes” to “always” in all the items (**Supplementary Table 2**; **Figure 1A**).

**Table 3:** Scale-level psychometric properties of Peruvian-Spanish SAVE-6 and SAVE-9 among healthcare workers.

Psychometric properties	SAVE-9	SAVE-6	Suggested cutoff
**Cronbach’s *α***	0.85	0.85	≥0.7
**McDonald’s *ω***	0.85	0.86	≥0.7
**IRT internal consistency**	0.92	0.91	≥0.7
Model fits of confirmatory factor analysis			
***χ*** ^2^ **(*df*, *p*-value)**	33.78 (26, 0.141)	4.84 (9, 0.848)	Non-significant
**CFI**	0.99	1.00	>0.95
**TLI**	0.99	1.00	>0.95
**RMSEA**	0.04	<0.001	<0.08
**SRMR**	0.07	0.04	<0.08

#### 3.3.2 Peruvian-Spanish version of SAVE-6

**Figure 2:**
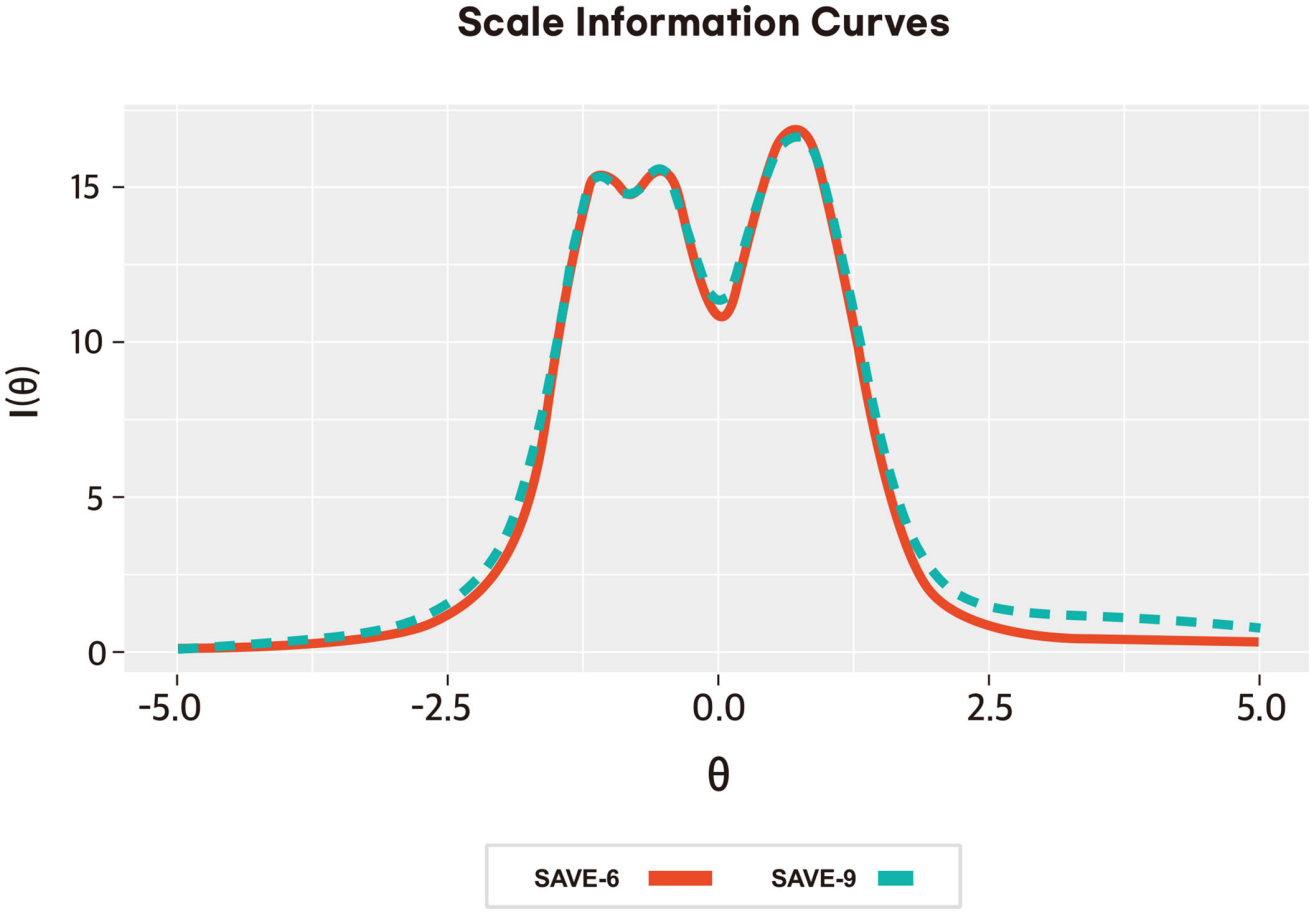
Scale information curve of the Peruvian-Spanish version of the SAVE-9 and SAVE-6 among healthcare workers.

The KMO value was 0.87 and Bartlett’s test of sphericity [*χ*2 (15) = 609.32, *p* < 0.001] showed that the sample was adequate and data were suitable for conducting factor analysis. Factor loadings ranged between 0.37 (item 4) and 0.89 (item 3) (**Table 2**). The CFA suggested that the Peruvian-Spanish SAVE-6 had good fit ([*χ* ^2^ (9) = 4.84, *p* = 0.848], CFI = 1.00, TLI = 1.00, RMSEA < 0.001, SRMR = 0.04) (**Table 3**). Multigroup CFA results showed invariance of the SAVE-6 across sex in metric or scale models (ΔCFI < 0.001) (**Supplementary Table 1**). In the GRM analysis, the items had a good fit (*p*-values of S-*χ* ^2^ at 0.62 and RMSEA values between <0.001 and 0.30) (**Supplementary Table 3**). The slope parameters (*a*) ranged between 0.78 (item 4) and 4.91 (item 3). The slope parameters of items 4 and 5 are moderate, and the rest are very high. The threshold parameter (*b*) results show that items 4 and 5 have much higher threshold values than the other items, implying that respondents need a much greater latent trait (*θ*) to endorse the highest response category for these questions. Items 1, 2, 3, and 8, on the other hand, have lower threshold values, indicating that they are significantly simpler to endorse across different levels of the latent trait (*θ*), with item 3 having the lowest (**Supplementary Table 3**; **Figure 1B**). **Figure 2** presents the scale information curve of SAVE-9 and SAVE-6. The scale information curves show that SAVE-9 and SAVE-6 are efficient to assess the latent trait between−2.25 and 2.25 theta level and provide similar levels of information.

### 3.4 Internal consistency of SAVE-9 and SAVE-6 and evidence based on relations to other variables

#### 3.4.1 Peruvian-Spanish version of SAVE-9

The Peruvian-Spanish SAVE-9 showed high internal consistency (Cronbach’s *α* = 0.85, McDonald’s *ω* = 0.85) and good criterion‐related validity based on Pearson’s correlation coefficient with GAD-7 (*r* = 0.44, *p* < 0.001) and PHQ-9 (*r* = 0.39, *p* < 0.001) scores. Based on the results from the ROC analysis, the appropriate cutoff point for SAVE-9 was calculated as ≥16 (area under the curve, AUC = 0.72, sensitivity = 0.75, specificity = 0.64) in accordance with a GAD-7 score ≥5 points.

#### 3.4.2 Peruvian-Spanish version of SAVE-6

The Peruvian-Spanish SAVE-6 showed high internal consistency (Cronbach’s *α* = 0.85, McDonald’s *ω* = 0.86) and good criterion‐related validity based on Pearson’s correlation coefficient with GAD-7 (*r* = 0.38, *p* < 0.001) and PHQ-9 (*r* = 0.35, *p* < 0.001) scores. Based on the results from the ROC analysis, the appropriate cutoff point for SAVE-6 was calculated as ≥11 (area under the curve, AUC = 0.72, sensitivity = 0.67, specificity = 0.63) in accordance with a GAD-7 score ≥5 points.

## 4 Discussion

In this study, we found that both Peruvian-Spanish SAVE-9 and SAVE-6 scales are reliable and valid rating scales for assessing epidemic-related anxiety in HCWs, specifically in response to the viral epidemic.

Confirmatory factor analysis demonstrated a good fit for the two-factor model of Peruvian-Spanish SAVE-9, similar to the original scale (14): factor I (items 1, 2, 3, 4, 5, and 8) and factor II (items 6, 7, and 9). This clustering was parallel to the other validation studies of Italian (17), Japanese (16), Turkish (19), and Malaysian (23) versions of the SAVE-9 scale. However, that clustering was not observed in other languages such as Russian (15) and German (18): factor I (items 2, 3, 4, and 8) and factor II (items 1, 5, 6, 7, and 9). Consistency with other languages suggests some universality in the factor structure, but differences in Russian and German indicate that anxiety perception may vary culturally (60). Regarding the SAVE-6 scale, we observed a good fit of the model for the unique structure. Originally, SAVE-6 was designed as a rating scale to assess the anxiety response of the general population (24), and it could also be applied to measure the anxiety response of HCWs. This finding is consistent with a study conducted in Spain that evaluated the validity and internal consistency of SAVE-6 on HCWs (25). Its adaptability for HCWs may be attributed to similarities in the experience of anxiety across different population groups.

The graded response model demonstrated that all items of SAVE-9 provided substantial information about work-related stress and anxiety in HCWs in response to viral epidemics. The threshold parameters reveal that these two items, 4 and 5, are considerably more demanding in terms of the latent trait required for endorsing the highest response category. Regarding the items’ performance, all items of SAVE-9 performed almost similar patterns to the Japanese (16) and Malaysian (23) versions. For SAVE-6, most items in this analysis exhibit very high slope parameters, suggesting a high ability to discriminate between different levels of the latent trait. The threshold parameters provide additional insights, showing that items 4 and 5 demand a significantly higher latent trait level for respondents to endorse the highest response category, signifying their relative difficulty. Scale information curves suggested that both scales provide similar levels of information about the latent traits; therefore, SAVE-6 has superiority over SAVE-9 in this regard.

The Peruvian-Spanish SAVE-9 and SAVE-6 also exhibited strong internal consistency and criterion‐related validity when compared with other anxiety scales like GAD-7 and PHQ-9. The internal consistency of the scales, based on Cronbach’s *α* and McDonald’s *ω*, was comparable to that of previous studies (18, 23, 25, 26). Based on the ROC analysis, the appropriate cutoff point for SAVE-9 was ≥16 in accordance with the 5 points of the GAD-7 (mild degree of general anxiety). Prior research reported cutoff scores for the SAVE-9 scale ranging from 14 to 22 in different study samples (14, 15, 18). Similarly, the appropriate cutoff point for SAVE-6 was calculated as ≥11 in accordance with a GAD-7 score ≥5 points. In previous studies, 12–16 points of cutoff scores for the SAVE-6 scale were reported (22, 24, 27, 28). Although the mental health questionnaires’ psychometric data and intercorrelations were comparable to those from previous samples, the reported levels of anxiety, as shown by the mean assessments, varied. Our sample exhibits lower scores on SAVE-9, SAVE-6, PHQ-9, and GAD-7. The timing of the study could account for this variation, as our data were collected between April and July 2023, despite the COVID-19 viral epidemic caused by the EG.5 strain (5). Dengue cases peaked during those months (7), and the transmission among patients and HCWs presented a heightened challenge although it was not deemed impossible (61, 62). Various factors could have contributed to the observed decrease in stress and anxiety levels among HCWs, including the widespread vaccination efforts.

While SAVE-9 addresses both anxiety and epidemic-specific stress, SAVE-6 focuses more directly on anxiety. This conceptual difference underscores the importance of examining both models to capture the full range of psychological responses to viral epidemics among healthcare workers. Opting to compare these scales sheds light on the nuanced facets of epidemic-related psychological distress, offering a comprehensive understanding that a singular model might not fully convey.

This study has several limitations. First, we used convenience sampling which could introduce selection bias, as the participants were selected so that they would be readily available at their workplace, which may not adequately represent HCWs. Second, although the sample size needed to conduct CFA could be achieved (32), the IRT requires larger samples (53), which may limit the ability of the study to detect significant effects. Finally, we chose to collect data in a specific period, from May to July 2023, and it is essential to clarify that the HCWs’ responses on the SAVE-9 and SAVE-6 scales may reflect their perceptions of stress and anxiety related to their most immediate experiences with viral outbreaks, including the context of the EG.5 strain of COVID-19 mentioned. Given the study’s cross-sectional design, we acknowledge that it captures a snapshot of HCWs’ mental health status within this specific timeframe. Therefore, while the study aims to assess the impact of viral epidemics on HCWs, it does not distinguish between current and past experiences of anxiety directly. The design does not allow for the direct assessment of previous anxiety levels before the study period. As such, interpretations of the SAVE-9 and SAVE-6 scale outcomes should consider that they represent HCWs’ self-reported anxiety and stress levels about their professional experiences during a period of heightened alert, without presupposing the absence or presence of prior anxiety conditions.

In the present study, the psychometric properties of SAVE-9 and SAVE-6 were compared. These scales had adequate item discrimination indices in both classical test theory and IRT. Additionally, its factor structure in Spanish is the same as that of other reported studies. These scales demonstrated good internal consistency and validity. The multigroup CFA suggested that this tool assesses the same construct across gender groups. These scales will be useful for researchers to measure anxiety among healthcare workers in Peru.

## Data availability statement

The raw data supporting the conclusions of this article will be made available by the authors, without undue reservation.

## Ethics statement

The studies involving humans were approved by Institutional Research Ethics Committee of the University of Piura. The studies were conducted in accordance with the local legislation and institutional requirements. The participants provided their written informed consent to participate in this study.

## Author contributions

BG: Conceptualization, Writing – original draft, Data curation, Methodology, Resources. RQ-L: Conceptualization, Data curation, Methodology, Writing – original draft, Resources. NJ-M: Conceptualization, Methodology, Writing – original draft. AL-R: Conceptualization, Methodology, Writing – original draft. FP-F: Conceptualization, Writing – original draft, Methodology. VV-R: Conceptualization, Methodology, Writing – review & editing. CA-D: Conceptualization, Methodology, Writing – review & editing. GV-G: Conceptualization, Methodology, Resources, Writing – review & editing. OA: Conceptualization, Formal analysis, Methodology, Writing – review & editing. SC: Conceptualization, Formal analysis, Methodology, Resources, Writing – review & editing, Visualization.
