# Supplementary material for: Comparison of the Stress and Anxiety to Viral Epidemic-9 and SAVE-6 scales among healthcare workers in Peru
Source: Front Psychiatry. 2024 May 1;15:1352896. doi: 10.3389/fpsyt.2024.1352896 (PMC11094251; doi:10.3389/fpsyt.2024.1352896)
Supplement: Supplementary file 1 [file Table_1.docx]

Supplementary Material

Comparison of the Stress and Anxiety to Viral Epidemic-9 (SAVE-9) and SAVE-6 Scales to measure anxiety among healthcare workers in Peru.

**Supplementary material**

**Appendix A. The original English version and the Peruvian Spanish version of the SAVE-9.**

| **Item** | **The Original English Version** | **The Peruvian Spanish Version** |
| --- | --- | --- |
| Item 1 | Are you afraid the virus outbreak will continue indefinitely? | ¿Le preocupa que la epidemia causada por el virus se prolongue indefinidamente? |
| Item 2 | Are you afraid your health will worsen because of the virus? | ¿Le preocupa que su salud empeore a causa del virus? |
| Item 3 | Are you worried that you might get infected? | ¿Le preocupa la posibilidad de infectarse? |
| Item 4 | Are you more sensitive towards minor physical symptoms than usual? | ¿Está más sensible de lo habitual a síntomas físicos menores? |
| Item 5 | Are you worried that others might avoid you even after the infection risk has been minimized? | ¿Le preocupa que los demás le eviten incluso cuando el riesgo de infección sea mínimo o haya desaparecido? |
| Item 6 | Do you feel skeptical about your job after going through this experience? | ¿Se cuestiona su trabajo después de pasar por esta experiencia? |
| Item 7 | After this experience, do you think you will avoid treating patients with viral illnesses? | Después de esta experiencia, ¿piensa que evitará tratar pacientes infectados por el virus? |
| Item 8 | Do you worry your family or friends may become infected because of you? | ¿Le preocupa infectar a familiares y amigos? |
| Item 9 | Do you think that your colleagues would have more work to do due to your absence from a possible quarantine and might blame you? | ¿Piensa que en el caso de una baja laboral por una posible cuarentena sus compañeros tendrían más trabajo y podrían culparle de ello? |

**Appendix B. The original English version and the Peruvian Spanish version of the SAVE-6.**

| **Item** | **The Original English Version** | **The Peruvian Spanish Version** |
| --- | --- | --- |
| Item 1 | Are you afraid the virus outbreak will continue indefinitely? | ¿Le preocupa que la epidemia causada por el virus se prolongue indefinidamente? |
| Item 2 | Are you afraid your health will worsen because of the virus? | ¿Le preocupa que su salud empeore a causa del virus? |
| Item 3 | Are you worried that you might get infected? | ¿Le preocupa la posibilidad de infectarse? |
| Item 4 | Are you more sensitive towards minor physical symptoms than usual? | ¿Está más sensible de lo habitual a síntomas físicos menores? |
| Item 5 | Are you worried that others might avoid you even after the infection risk has been minimized? | ¿Le preocupa que los demás le eviten incluso cuando el riesgo de infección sea mínimo o haya desaparecido? |
| Item 6 | Do you worry your family or friends may become infected because of you? | ¿Le preocupa infectar a familiares y amigos? |

**Supplementary Table 1. Measurement invariance of the SAVE-6 and SAVE-9 scales across male and female.**

| **Model** | **χ^2^** | **df** | **Δ χ^2^** | **Δdf** | **P** | **CFI** | **ΔCFI** |
| --- | --- | --- | --- | --- | --- | --- | --- |
| **SAVE-9; Sex (male vs. female)** | | | | | | | |
| **Configural** | 46.273 | 52 |  |  |  | 1.000 |  |
| **Metric** | 53.295 | 59 | 7.022 | 7 | 0.427 | 1.000 | .000 |
| **Scalar** | 61.422 | 66 | 8.127 | 7 | 0.322 | 1.000 | .000 |
| **SAVE-6; Sex (male vs. female)** | | | | | | | |
| **Configural** | 7.161 | 18 |  |  |  | 1.000 |  |
| **Metric** | 13.289 | 23 | 6.128 | 5 | 0.294 | 1.000 | 0.000 |
| **Scalar** | 20.353 | 28 | 7.064 | 5 | 0.216 | 1.000 | 0.000 |
| CFI = comparative fit index | | | | | | | |

**Supplementary Table 2. Graded response model output of the SAVE-9 scale.**

| **Items** | **Item fits** | | | | **Slope parameter (a)** | **Threshold parameter (b)** | | | |
| --- | --- | --- | --- | --- | --- | --- | --- | --- | --- |
|  | **S-χ^2^** | **df** | **p value** | **RMSEA** |  | **b_1_** | **b_2_** | **b_3_** | **b_4_** |
| **Factor I** | | | | | | | | | |
| **Item 1** | 17.541 | 20 | .618 | .000 | 3.710 | -1.083 | -.478 | .509 | 1.121 |
| **Item 2** | 23.623 | 20 | .618 | .030 | 3.719 | -1.176 | -.389 | .496 | 1.016 |
| **Item 3** | 15.743 | 15 | .618 | .016 | 4.910 | -1.145 | -.497 | .550 | .878 |
| **Item 4** | 28.511 | 28 | .618 | .009 | .780 | -2.324 | .426 | 3.251 | 5.747 |
| **Item 5** | 27.433 | 29 | .618 | .000 | 1.040 | -1.752 | .525 | 2.414 | 3.697 |
| **Item 8** | 23.905 | 24 | .618 | .000 | 2.207 | -1.663 | -.885 | .187 | .959 |
| **Factor II** | | | | | | | | | |
| **Item 6** | 10.944 | 8 | .407 | .043 | 1.863 | -.490 | .671 | 2.071 | 2.676 |
| **Item 7** | 8.545 | 7 | .407 | .033 | 1.732 | -.133 | 1.030 | 2.201 | 2.669 |
| **Item 9** | 11.438 | 11 | .407 | .014 | .720 | -1.549 | .439 | 2.663 | 4.462 |
| RMSEA: Root mean square error of approximation | | | | | | | | | |

**Supplementary Table 3. Graded response model output of the SAVE-6 scale.**

| **Items** | **Item fits** | | | | **Slope parameter (a)** | **Threshold parameter (b)** | | | |
| --- | --- | --- | --- | --- | --- | --- | --- | --- | --- |
|  | **S-χ^2^** | **df** | **p value** | **RMSEA** |  | **b_1_** | **b_2_** | **b_3_** | **b_4_** |
| **Item 1** | 17.541 | 20 | .618 | .000 | 3.710 | -1.083 | -.478 | .509 | 1.121 |
| **Item 2** | 23.623 | 20 | .618 | .030 | 3.719 | -1.176 | -.389 | .496 | 1.016 |
| **Item 3** | 15.743 | 15 | .618 | .016 | 4.910 | -1.145 | -.497 | .550 | .878 |
| **Item 4** | 28.511 | 28 | .618 | .009 | .780 | -2.324 | .426 | 3.251 | 5.747 |
| **Item 5** | 27.433 | 29 | .618 | .000 | 1.040 | -1.752 | .525 | 2.414 | 3.697 |
| **Item 8** | 23.905 | 24 | .618 | .000 | 2.207 | -1.663 | -.885 | .187 | .959 |
| RMSEA: Root mean square error of approximation | | | | | | | | | |
